# 5HT2A modulation attenuates pancreatic cancer induced pain mouse model by inhibiting HDAC

**DOI:** 10.1590/acb392324

**Published:** 2024-04-15

**Authors:** Weiwei Fan, Xijia Yang, Liang Zhou, Jianqing Xu, Weihua Huang, Alok Shiomurti Tripathi

**Affiliations:** 1 Xi’an Gaoxin Hospital – Department of General Surgery – Xi’an – China.; 2 ERA University – ERA College of Pharmacy – Department of Pharmacology – Lucknow – India.

**Keywords:** Pancreatic Neoplasms, Pain, Serotonin, Histone Deacetylases, Cytokines

## Abstract

**Purpose:**

Patients have been severely suffered from cancer associated pain, and pancreatic cancer is the most severe form of cancer associated with pain. There are very few options available to manage it. The present report evaluated the effect of 5HT2A on pancreatic cancer associated pain.

**Methods:**

Pancreatic cancer was induced by injecting SW 1,990 cells (~3×10^6^in a 20 μL suspension) into the pancreas and formed a 2–3-mm vesicle using an inoculator fitted with a 26-gauge needle in BALB/c-nu mice. Survival rate and body weight of the mice were observed. Pain behaviour testing was performed at the end of each week (third and fourth week) after surgery. Inflammatory mediators and HDAC 2 proteins were determined in the spinal tissue using quantitative real-time polymerase chain reaction.

**Results:**

There was improvement in the survival rate and body weight in 5HT2A antagonist treated group than pancreatic cancer group of mice. Moreover, 5HT2A antagonist ameliorated the alteration in pain behaviour of pancreatic cancer mice. mRNA expression of HDAC2 and level of inflammatory cytokines were reduced in the spinal tissue of 5HT 2A antagonist treated group than pancreatic cancer group of mice.

**Conclusions:**

Data revealed that 5HT2A antagonist ameliorates pain associated with pancreatic cancer mice by HDAC inhibition and inflammatory cytokines. The result of investigation supports that modulation of 5HT2A receptor could be used clinically to protects neuropathic pain in pancreatic cancer.

## Introduction

Cancer is one of the major diseases and affects approximately 15 million people throughout the globe^1^. Several reports suggest that prevalence of cancer associated pain is observed to be up to 90%^2^. Quality of life impairs, and survival rate affectes patients suffering from cancer due to associated pain with it^3^. Conventional therapies available for the management of cancer associated pain have several limitations. Thus, there is the need of a specific treatment to manage it.

There are several pathways involved in the pain associated with pancreatic cancer, such as histone acetylation^4^. Histone deacetylases (HDACs) and histone acetyltransferases (HATs) regulate the acetylation of histone^5^. HDAC alters the gene expression as it closes the conformation of chromatin, deacylated the DNA^6^. Literature reveals that inhibition of HDAC shows analgesic effect after intrathecally or systemically administration HDAC inhibitors in rodents^7^. Endogenous analgesic effect occurs due to activation of μ-opioid receptor, which is altered due to the modification of histone^8^. Moreover, HDAC reported to have role in pain associated with chronic pancreatitis.

5HT2A is one of the receptors of 5-hydroxytryptamine, a neurotransmitter reported to be involved in the regulation of several physiological function^9^. Its antagonism is involved in the regulation of pain related behavior. Moreover, 5HT2A antagonist reported to ameliorate the neurogenic pain in several neurological related disease^10^. Thus, the present report evaluated the effect of 5HT2A antagonist against neuropathic pain associated with pancreatic cancer.

## Methods

### Chemicals

5HT2A antagonist, i.e., ketanserin, was procured from DuPont NEN Research Products, United States of America. Enzyme-linked immunosorbent assay (ELISA) kit of tumor necrosis factor (TNF)-α, interleukin (IL)-1β and IL-6 were purchased from ThermoFisher Scientific Ltd., United States of America. HDAC2 and other primers were procured from Sino Biologicals, Beijing, China.

### Animals

BALB/c-nu mice (Female; 20–38 g) were acclimatized in an animal house facility for a week. All the animals were maintained at 23 ± 2°C, 55 ± 5% humidity, in a natural light and dark cycle, with free access to food and water and pellet diet ad libitum. The experimental protocols were approved by institutional animal ethical committee (650/02/C/CPCSEA/01).

### Experimental

All the mice were separated into three different groups: control group; negative control group; and 5HT2A antagonist treated group, which received ketanserin (1 mg/kg s.c.)^10^. All the mice were exposed to isoflurane for the induction of anesthesia, and an incision was made to expose the spleen and pancreas after opening the abdomen. SW 1,990 cells (~3×10^6^in a 20-μL suspension) were administered into pancreas slowly with a 26-gauge needle fitted inoculator to form 2–3-mm vesicle^11^. Suture was performed to the incision site after placing the organ again, and each animal was kept separately. While sham was operated, mice received equal volume of medium.

### Assessment of body weight and survival rate

Body weight of each mouse was estimated at the end of each week after surgery for four weeks. Moreover, survival rate was analyzed among different groups.

### Assessment of pain behavior

Pain behavior was assessed in pancreatic cancer mice as previously reported method^12^. Mechanical sensitivity of abdomen was indicated with withdrawal thresholds to mechanical stimuli. von Frey apparatus was used to determine the mechanical threshold. Stimulus was applied to the epigastric region, and withdrawal of abdomen was considered as positive response.

### Assessment of cytokine level

Spinal cord tissue isolated from each mouse was homogenized under phosphate buffer, and levels of TNF-α, IL-1β and IL-6 were estimated in the tissue homogenate of pancreatic cancer mice model using ELISA method.

### Quantitative real-time polymerase chain reaction

Spinal cord tissue isolated from each animal was homogenate, and total RNA isolation system was used to extract RNA from these tissues. Moreover, PrimerScript RT reagent kit was used to reverse transcription of mRNA to cDNA. Real-time polymerase chain reaction (PCR) instrument was utilized with Real Master Mix Kit to perform RT-PCR reactions. The reaction was performed for 30 s at 95°C; 5 s at 95°C; and 40 cycles at 60°C × 20 s, and thereafter 30 s at 60 °C. Melt curve analysis was carried out at the temperature to 95°C. Relative expression of gene results were expressed as relative expression of gene compared to glyceraldehyde 3-phosphate dehydrogenase (GAPDH), and primers used in present study were represented as follows:

**Table:** 

Sr. No.	Primer	Forward	Reverse
1.	HDAC2	5’-GCTGCTTCAACCTAACTG-3’	5’-CTCATACGTCCAACATCG-3’
2.	GAPDH	5’-CCTGGAGAAACCTGCCAAG-3’	5’-CACAGGAGACAACCTGGTCC-3’

### Statistical analysis

Results were expressed as mean ± standard error of the mean (n = 20). The statistical analysis was performed using one-way analysis of variance (ANOVA) followed by Dunnett’s test for multiple comparisons (GraphPad Prism software, version 6.1; United States of America). The level of statistical significance was set at P < 0.05.

## Results

### Effect of 5HT2A antagonist on survival rate

**Figure 1:**
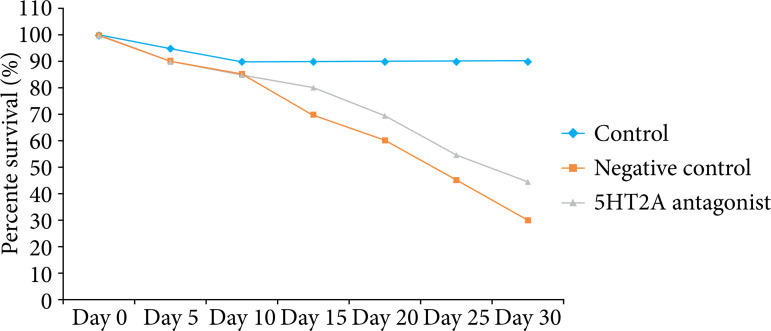
Effect of 5HT2A antagonist on percent of survival in pancreatic cancer mice.

Source: Elaborated by the authors.

Percent of survival was estimated for 5HT2A antagonist treated pancreatic cancer mice model after four weeks of surgery, as shown in Fig. 1. Percent survival of negative control group mice was observed up to 30%, which is lower than control group, i.e., 90% at the fourth week after surgery. Moreover, treatment with 5HT2A antagonist improved survival rate marginally up to 45% until the end of protocol.

### Effect of 5HT2A antagonist on body weight

**Figure 2:**
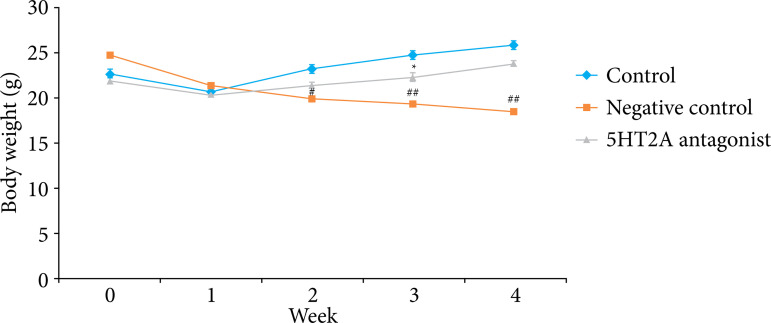
Effect of 5HT2A antagonist on body weight of pancreatic cancer mice.

Body weight of 5HT2A antagonist treated pancreatic cancer mice was estimated on every week until the fourth week after surgery, as shown in Fig. 2. There was significant reduction in body weight of negative control group than control group of rats. Treatment with 5HT2A antagonist improved the body weight of pancreatic cancer mice.

Mean ± standard error of the mean (n = 20); ^#^p<0.05, ^##^p < 0.01 compared to control group; *p < 0.05, **p < 0.01 compared to negative control group. Source: Elaborated by the authors.

### Effect of 5HT2A antagonist on pain behavior

**Figure 3:**
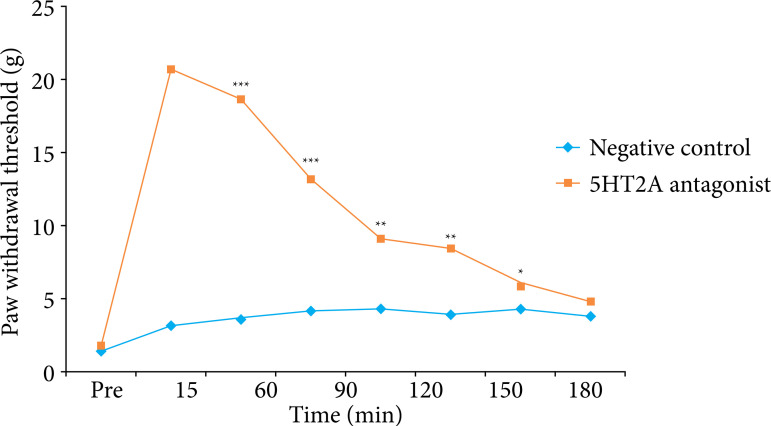
Effect of 5HT2A antagonist on paw withdrawal threshold in pancreatic cancer mice.

Analgesic effect 5HT2A antagonist was estimated by determining paw withdrawal threshold in pancreatic cancer mice using von Frey apparatus, as shown in Fig. 3. Paw withdrawal threshold was estimated at 15, 30, 60, 90, 120, 150 and 180 min after the administration of 5HT2A antagonist. There was significant increase (p < 0.001) in the paw withdrawal threshold in 5HT2A treated group than negative control group.

Mean ± standard error of the mean (n = 20); *p < 0.05, **p < 0.01, ***p < 0.001 compared to negative control group. Source: Elaborated by the authors.

### Effect of 5HT2A antagonist on inflammatory cytokines

**Figure 4:**
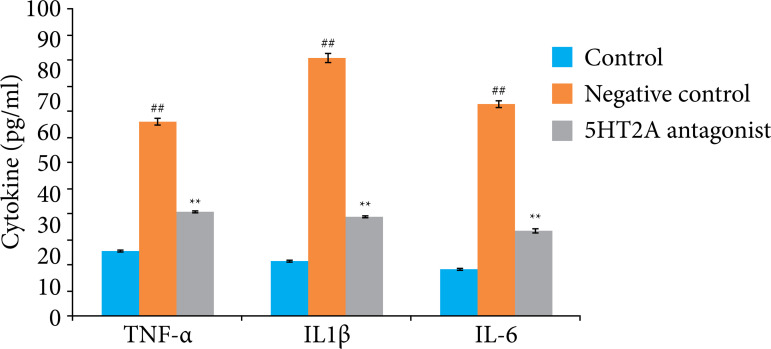
Effect of 5HT2A antagonist on level of inflammatory cytokines in spinal tissue of pancreatic cancer mice

Inflammatory cytokines like TNF-α, IL-1β and IL-6 were estimated in the spinal tissue of 5HT2A antagonist treated pancreatic cancer mice. Levels of TNF-α, IL-1β and IL-6 in inflammatory cytokines were enhanced significantly (p < 0.01) in spinal tissue homogenate of negative control group than control group of mice. There was significant reduction in inflammatory cytokines in spinal tissue of 5HT2A antagonist treated group compared to the negative control group of mice (Fig. 4).

Mean ± standard error of the mean (n = 20); ^##^p < 0.01 compared to control group; **p < 0.01 compared to negative control group; TNF: tumor necrosis factor; IL: interleukin. Source: Elaborated by the authors.

### Effect of 5HT2A antagonist on expression of HDAC

**Figure 5:**
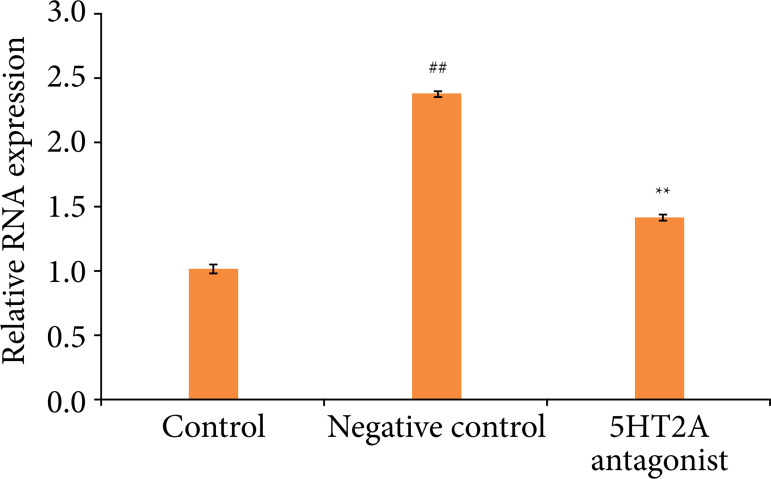
Effect of 5HT2A antagonist on relative mRNA expression of HDAC2 in spinal cord tissue homogenate of pancreatic cancer mice.

Expression of HDAC2 was estimated in the spinal tissue homogenate of 5HT2A antagonist treated pancreatic cancer mice, as shown in Fig. 5. There was significant increase (p < 0.01) in the mRNA expression of HDAC in tissue homogenate of negative control group than control group of mice. mRNA expression of HDAC was reduced in spinal tissue of 5HT2A antagonist treated group than negative control group of mice.

Mean ± standard error of the mean (n = 20); ^##^p < 0.01 compared to control group; **p < 0.01 compared to negative control group. Source: Elaborated by the authors.

## Discussion

Pancreatic cancer reportedly induces neuropathic pain, which reduces the quality of life. Tissue injury and diseased condition were slow down by reducing nociceptive response^13^. Pain associated with early or late-stage pancreatic cancer is effectively managed with endogenous central nervous system (CNS) opioid analgesic^14^. However, chronic administration of these conventional drugs has several limitations.

Thus, the present investigation evaluated the analgesic effect of 5HT2A antagonist against pancreatic cancer mice. Data revealed that survival rate was reduced in negative control group than control group of mice, which represents that pancreatic cancer associated pain contributes to the progression of disease^15^, and the management of pain associated with pancreatic cancer ameliorates the condition. There was significant improvement in the survival rate in 5HT2A antagonist treated pancreatic cancer mice.

Pain associated with pancreatic cancer occurs in the highly advanced stage of tumor, and in humans pain radiates from epigastric area to back^16^. Pain threshold reduces due to visceral pain, which occurs due to mechanical stimulation of abdominal skin^17^. Pain associated pancreatic cancer reported to be manage with the administration CNS related drug, which reported to regulate behavioral response in animal^18^. The present report reveals that treatment with 5HT2A antagonist improves mechanical threshold in pancreatic cancer mice. Moreover, weight loss is also prevented in 5HT2A antagonist treated group compared to negative control group mice.

Inflammatory cytokines were reported to contribute to the development of cancer and cancer associated pain, including pancreatic cancer^19^. Pain relates with inflammation. There are several mechanisms involved in the development of pain, such as ischemia and compression of nerve or increase in the level of cytokines^20^. Data of the reported study supports it, and treatment with 5HT2A antagonist significantly (p < 0.01) reduces the level of inflammatory cytokines in the spinal tissue compared to negative control group of mice.

HDAC inhibitors have emerged as a role in regulation of synaptic and neuroplastic activity, also contributing to reducing neuroinflammation and neuronal damage^21^. Neuropathic pain in animal model reported to be reduced with HDAC inhibitors^22^. HDAC inhibition also regulates the modulation of pancreatic cancer^4^. The present report suggests that treatment with 5HT2A antagonist significantly (p < 0.01) reduces mRNA expression of HDAC in the spinal tissue of pancreatic cancer mice.

## Conclusion

In conclusion, data of this report revealed that treatment with 5HT2A antagonist improves the survival rate and ameliorates pain threshold in pancreatic cancer mice, as it significantly reduces the level of inflammatory cytokines and mRNA expression of HDAC inhibition and inflammatory cytokines. The result of the investigation supports that modulation of 5HT2A receptor could be used clinically to protects neuropathic pain in pancreatic cancer.

## Data Availability

All data generated or analyzed during this study are included in this article.
